# Case series of the inferior vena cava primary leiomyosarcoma treatment

**DOI:** 10.1093/jscr/rjad546

**Published:** 2024-06-05

**Authors:** Davidovic Lazar, Ducic Stefan, Dragas Marko, Petar Zlatanovic, Milos Sladojevic, Cinara Ilijas, Nikica Grubor, Dimic Andreja

**Affiliations:** Faculty of Medicine, University of Belgrade, Belgrade, Serbia; Clinic for Vascular and Endovascular Surgery, University Clinical Center of Serbia, Koste Todorovića Street 8, Belgrade 11000, Serbia; Faculty of Medicine, University of Belgrade, Belgrade, Serbia; Faculty of Medicine, University of Belgrade, Belgrade, Serbia; Clinic for Vascular and Endovascular Surgery, University Clinical Center of Serbia, Koste Todorovića Street 8, Belgrade 11000, Serbia; Faculty of Medicine, University of Belgrade, Belgrade, Serbia; Faculty of Medicine, University of Belgrade, Belgrade, Serbia; Clinic for Vascular and Endovascular Surgery, University Clinical Center of Serbia, Koste Todorovića Street 8, Belgrade 11000, Serbia; Faculty of Medicine, University of Belgrade, Belgrade, Serbia; Faculty of Medicine, University of Belgrade, Belgrade, Serbia; Clinic for Digestive Surgery, University Clinical Center of Serbia, Belgrade, Serbia; Faculty of Medicine, University of Belgrade, Belgrade, Serbia; Clinic for Vascular and Endovascular Surgery, University Clinical Center of Serbia, Koste Todorovića Street 8, Belgrade 11000, Serbia

**Keywords:** leiomyosarcoma, inferior vena cava, reconstruction, complete resection

## Abstract

Tumors of the inferior vena cava (IVC) are rare and usually malignant and they can be primary and secondary. The most common primary tumor of the IVC is primary leiomyosarcoma. The first case of primary IVC leiomyosarcoma has been described in 1871 [1].The total number of 218 cases has collected until 1996 [2]. After that, three large single center series of these tumors emerged [3–5]. Present a series of five cases of these tumors. All the patients underwent a wide complete resection of tumors and the reconstruction with Dacron grafts. One patient died 19 months after the surgery, while the remaining ones survived without a local and system disease relapse. Although a surgical resection combined with the chemotherapy is often not curative, it can achieve a significant long-term survival. For this reason, we recommend the aggressive surgical management using the modern vascular surgical and oncology techniques.

## Case series

### Study design

This was a single-center retrospective study that evaluated the medical records of patients undergoing surgical resection of the primary leiomyosarcoma (LMS) of inferior vena cava (IVC) in the Clinic for vascular and endovascular surgery, Clinical Center of Serbia in the period from January 2014 to March 2021.All procedures were in accordance with the ethical standards of the responsible committee on human experimentation (institutional and national) and with the Helsinki Declaration of 1964 and its later amendments. Informed consent was obtained from all patients included in the study.

## Result

Overall, five patients were diagnosed with primary LMS of the IVC at our institution from 2014 to 2021. All of our patients were female. The median age of diagnosis was 53 years (range, 51–74). Comorbidities were notable for hypertension in four patients (80%) and diabetes mellitus in one patient (20%). The median body mass index (BMI) was 25.9 kg/m^2^ (range, 22.8–28 kg/m^2^). In two patients, the main symptom of the presentation is pain in the hip / back (40%), where one patient even underwent an exploratory laparotomy due to suspicion of an acute abdomen. The other three patients (60%) reported nonspecific symptoms or the tumor was discovered incidentally on systematic examination. (Table 1).

**Table 1:** Baseline, perioperative, operative, and postoperative data patients with LMS of IVC.

Baseline characteristics and demographic data	*n* = 5 (%)
Female	5 (100%)
Age	52 (IQR, 44–63)
Arterial hypertension	4 (80%)
BMI	(range, 22.8–28 kg/m^2^)
DM	1 (20%)
Symptoms on presentation	
Abdominal pain/ back pain	2 (40%)
Nonspecific symptoms	3 (60%)
IVC segment involved	
Lower	2 (40%)
Middle	3 (60%)
Upper	0 (0%)
Operative data	
Type of reconstruction	
Dacron graft	5 (100%)
Operation performed	
Graft interposition	2 (40%)
Graft interposition and reimplantation of hepatic/renal vein	3 (60%)
Additional organ resection	1 (20%)
Total operative time	212
Blood loss (ml)	1126 (IKR 450–1125)
Postoperative outcome	
Pneumothorax	1 (20%)
Hospital mortality	0 (0%)

**Figure 1:**
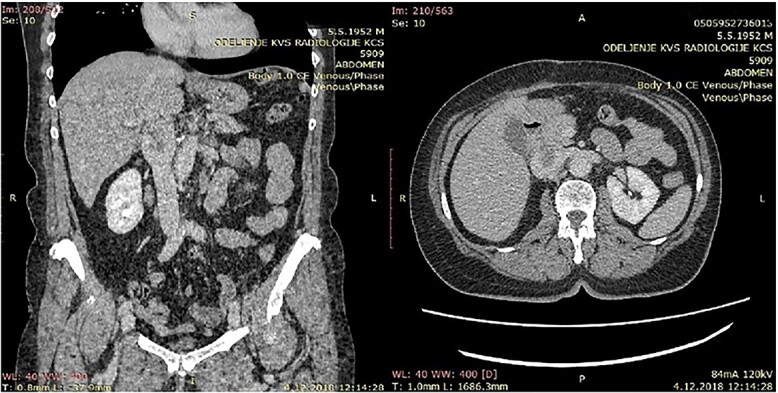
CT of the abdomen that shows a retroperitoneal tumor mass that involves IVC above the renal and under the liver veins.

**Figure 2:**
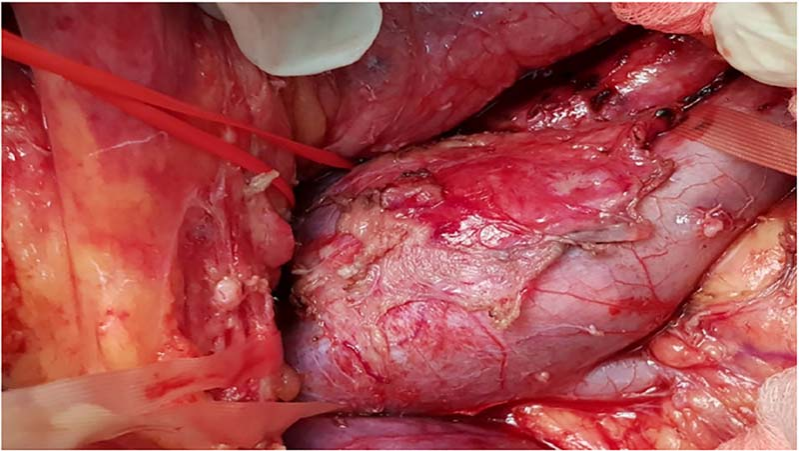
Intra-operative photo showing tumor involve infrarenal segment IVC and level of renal veins.

**Figure 3:**
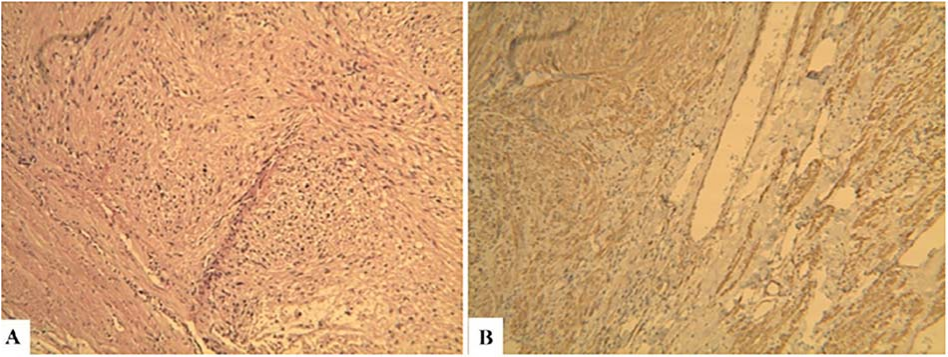
Histopathological findings. Microscopic examination revealed that the tumor consisted of uniform and spindle cells and had a fascicular growth pattern (Hematoxylin & Eosin: ×600) (A). Immunohistochemically staining revealed that α-SMA positive (×600) (B).

**Figure 4:**
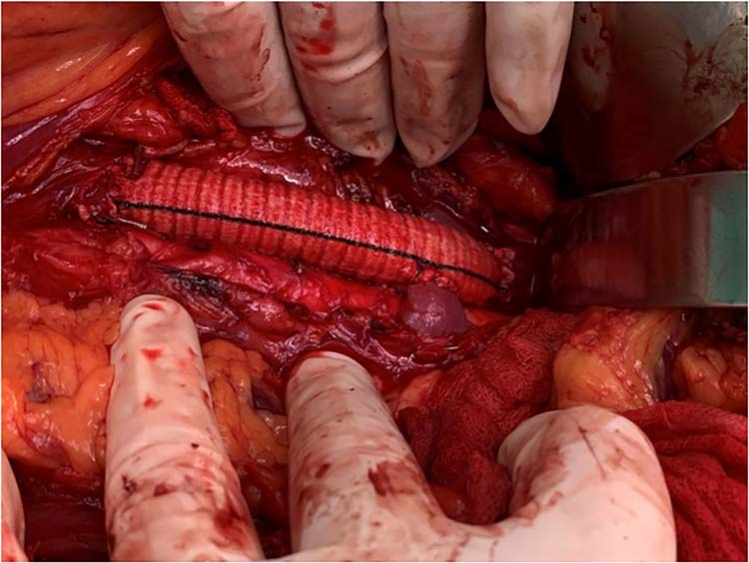
View of the reconstruction IVC with 22 mm Dacron graft and reimplantation left renal vein directly to the graft.

All five patients underwent surgical resection of primary LMS. A majority of patients had involvement of only their suprarenal IVC (three patients; 60%). Two patients (40%) had tumor involving infrarenal segment IVC (I segment). IVC was reconstructed using Dacron grafts in all patients (five patients; 100%). A bypass was done on the left hepatic vein of one patient, while the other two patients required reimplantation of the renal veins directly into the graft (while one of them, the other to the left of the RV). Additional organ resection was required in one patient (20%): in whom cholecystectomy was performed and partial liver resection due to metastasis in the left lobe of the liver. The mean total duration of surgery was 212 minutes, and the average estimated blood loss was 1126 ml.

All five patients (100%) were admitted to the intensive care unit of the postoperative care unit and the total mean length of the hospital the stay was 8 days (IKR, 6–13 days). One patient had pneumothorax. There was no 30-day mortality, and there was no occurrence of myocardial infarction, pulmonary embolism, wound infection, bleeding requiring surgical treatment, or deep vein thrombosis of the lower extremities. Tumor growth was found to be relatively evenly distributed with predominantly intraluminal in three cases (60%), and mixed in two cases (40%). The median tumor size was 5 cm (IQR, 4.5–9 cm). All but one patient had intermediate (grade 2) or high-grade (grade 3) tumor, based on FNCLCC criteria.

Our patients came for regular control examination, first a month after the operation, then for 6 months and then for a year. One patient died 19 months after surgery. It was a patient who had liver metastases, which were also removed during surgery. Other patients are alive, with no local or systemic signs of remission. A survival rate after mean follow-up of 41.4 months is 80%.

## Discussion

Primary LMS is rare, malignant tumor originating from the smooth muscle cells of the middle layer of the IVC [6–8].These tumors are more prevalent in women, and patients are mostly in their sixth decades [5, 8, 9]. All of our five patients were females with average age of 53 years (range, 51–74).

The site of origin of the primary LMS from the IVC is described in relation to the hepatic and renal veins. For this purpose, Kulaylat and associates divided the IVC into lower (infrarenal-I), middle (suprarenal-II), and upper (suprahepatic-III) segments [10]. The lower (infrarenal) segment extends from the confluence of the common iliac veins to the renal veins. The middle (suprarenal) segment extends from the renal to the hepatic veins, while the upper (suprahepatic) segment extends from the hepatic veins up to the right atrium. The middle (suprarenal) IVC is further classified into infra- and retrohepatic portions, based on the relationship to the caudate lobe veins [10]. The percentages of the upper (suprahepatic), middle (surarenal), and lower (infrarenal) IVC segment involvement with primary LMS are 24, 42, and 34%, respectively [5, 9, 11]. In our study, three (60%) of primary LMS involved suprarenal, while two (40%) infrarenal segment of the IVC. A total of 62% of these tumors demonstrate extraluminal, 5% intraluminal, and 33% extra and intraluminal growth patterns^5^. All five of our cases had extra and intraluminal growth patterns.

The clinical presentation of the primary IVC LMS varies according to their dimensions, growth pattern, and the localization. The upper section involvement might present with Budd–Chiari syndrome, middle section involvement with nephritic syndrome, and infrarenal involvement with lower extremity edema [12]. Patients generally present with nonspecific symptoms, such as exhaustion, abdominal pain, and weight loss [1–12]. Most of our patient had also nonspecific symptoms or the disease was accidentally discovered.

Because of previous reasons the diagnosis of the primary IVC LMS is often incidental. When symptoms are present, the computerized tomography (CT) or NMR are useful to confirm the presence of a tumor, its pattern of growth, relationship to the surrounding structures, and the presence of caval obstruction [10, 12]. In all five of our cases, MDCT angiography showed tumor of the IVC. The final ultimate diagnosis, of course, is carried out using histopathological and immunohistochemical methods. The pathognomonic findings of LMS are spindle-shaped tumor cells with positive markers for smooth muscle cells, vimentin, muscle actin, alpha-smooth-muscle actin, and desmin [13].

The recommended therapy for treating of primary IVC LMS is aggressive surgical removal of the tumor in combination with chemotherapy and/or radiotherapy [10, 14]. All our patients have undergone this type of treatment.

The surgical approach depends on the IVC segment affected by the tumor and need for additional major liver resection or cardiopulmonary bypass. The lower (infrarenal) segment of the IVC is approaching through midline laparotomy that is followed with medial rotation of the right colon and mesenteric base (two of our cases) [15]. An exposure of the middle (suprarenal) segment of the IVC requires a bilateral subcostal incision. The next step is medial rotation of the right colon, mesenteric base, the first duodenal portion (Kocher maneuver), as well as pancreas. This approach allows usually adequate exposure and an isolation of the infrahepatic IVC [15, 16]. We had three such cases.

However, an approach to the retrohepatic segment of the IVC requires additional procedures. After already explained approach of the infrahepatic IVC, the next step is a detachment of diaphragmatic peritoneum from the liver. This maneuver enables the identification of suprahepatic veins and the isolation of the suprahepatic IVC with surgical tape [17]. The procedure continues with the dissection of the IVC from the posterior margin of the liver through a careful section of all accessory hepatic veins. After complete overturning of the liver up and to the left, an IVC can be isolated from the diaphragm to the iliac confluence [17]. Veno-venous bypass may be needed only if the systolic blood pressure cannot be maintained over 100 mmHg during a test clamp of the suprahepatic IVC. Veno-venous bypass is required more often in patients with cardio - pulmonary dysfunction, or in those over age 50 years [15, 18].

The first surgical treatment of IVC LMS was performed in 1928 [19]. A radical excision followed with replacement of IVC is conventional standard for the treatment of the IVC primary LMS in contemporary practice [6, 7, 9, 15]. The obligatory replacement of resected suprarenal IVC is recommended because of the risk of renal dysfunction and lower extremity edema without a caval graft [16, 20, 21]. The decision to replace the infrarenal IVC during cancer resection is controversial [15, 16, 20–23]. According to guidelines of the American Venous Forum, the replacement of the infrarenal IVC is recommended if it was patent before surgery, if the collateral circulation appears inadequate following caval resection and in those in whom important collateral veins had to be ligated or resected during tumor removal [20]. Externally supported PTFE grafts are used most often for replacement of IVC [11, 15, 16, 23, 24]. However, Dacron graft is increasingly used in the reconstruction of IVC [25]. In all five of our cases, a Dacron graft was used for reconstruction after radical excision of the primary IVC LMS.

Patients underwent IVC replacement with prosthetic grafts require LWMH subcutaneously over the first three postoperative days. They should be dismisssed from the hospital on warfarin 6 months and aspirin after that [26, 27]. Lower extremity pneumatic compression is also recommended during the early postoperative period. This therapeutic scheme was used in all our patients. A role of adjuvant chemotherapy and radiation therapy is not well studied and is dependent on the judgment of the surgical and medical oncologists. Adjuvant therapy was not used in our cases.

A 5-year survival rate after radical excision of primary IVC LMS replacement ranges between 35 and 50% [7, 8, 26]. In our study, a survival rate after mean follow-up of 41.4 months is 80%.

## Conclusion

Primary LMS IVC is a challenging disease that requires well-coordinated multimodal therapy. Radical surgical treatment is the only option for patients with IVC LMS if they do not have significant comorbidities. In our series of cases, we have shown that despite the biologically aggressive nature of tumors, long-term survival can be achieved if a multidisciplinary team led by an experienced vascular surgeon treats these patients.

## Conflict of interest statement

None declared.

All authors read and approved the final version of the manuscript.
